# The enzymatic analysis of alcohol (ethanol) in serum and plasma with the alcohol dehydrogenase reagent: focus on intra-analytical and post-analytical aspects

**DOI:** 10.11613/BM.2025.030501

**Published:** 2025-08-15

**Authors:** Cristiano Ialongo, Alan Wayne Jones

**Affiliations:** 1 Department of Clinical Pathology, University Hospital Policlinico Umberto I, Rome, Italy; 2 Division of Clinical Chemistry and Pharmacology, Department of Biomedical and Clinical Sciences, Faculty of Medicine and Health Sciences, University of Linköping, Linkoping, Sweden

**Keywords:** blood alcohol content, alcoholic intoxication, substance abuse detection, human toxicology

## Abstract

The alcohol dehydrogenase (ADH) method is commonly used to measure serum alcohol concentration (SAC) and plasma alcohol concentration (PAC) for the rapid detection of ethanol intoxication in emergency medical departments. Alcohol dehydrogenase methods are sometimes used in forensic laboratories as a preliminary screening test prior to confirmation by gas chromatographic (GC) methods. This review identifies critical factors affecting results of ADH methods of analysis including clinical reliability and forensic defensibility. Key considerations include intra-analytical factors (method chemistry, calibration, analytical performance, interferences, calibrator stability, and sample matrix effects) and post-analytical factors (measurement units, reference ranges, performance specifications, uncertainty budget, medical decision levels, legal intoxication thresholds, ADH-GC agreement, and SAC/PAC to blood alcohol concentration (BAC) conversion). The yeast ADH method demonstrates high selectivity for ethanol with no assay-specific bias, and measurement error and uncertainty meet regulatory standards. However, ADH methods are prone to interferences, particularly from lactate dehydrogenase/lactic acid (LD/LA), leading to potential false positive results. Free hemoglobin (hemolysis) is another problem with ADH methods introducing a negative bias. When results provided by hospital laboratories are interpreted in a legal context, care is needed because ethanol concentrations in plasma/serum are about 15% higher than in whole blood (range 10-20%). Although less important in clinical practice, these differences are important to consider in a forensic context. The ADH method is not inherently a forensic assay, but these limitations can be mitigated by refining laboratory procedures and standardizing the assay methodology and quality control, thus strengthening forensic reliability and boosting confidence in the analytical results.

## Graphical abstract

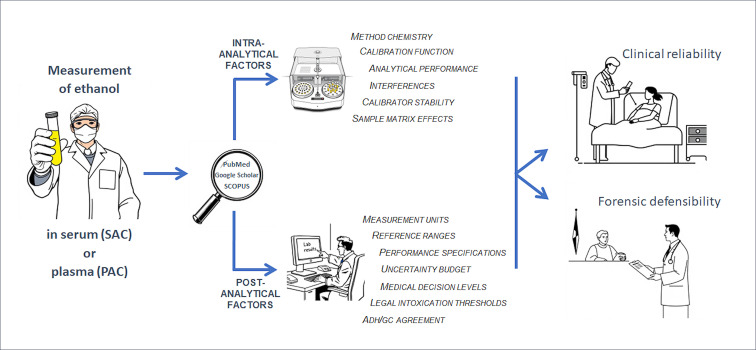

## Highlights

• Hospital laboratories measure ethanol in plasma or serum by enzymatic method

• Lactate dehydrogenase/lactic acid interference can cause false positive results

• The alcohol dehydrogenase (ADH) methods’ allowable error and measurement uncertainty meet regulatory standards

• ADH oxidation methods cannot replace gas chromatography methods in forensic analysis

• Laboratory procedures and assay standardization can mitigate method limitations

## Introduction

Ethanol (EtOH, CAS 64-17-5), also known as ethyl alcohol or just alcohol, has a long history in human culture and society, especially regarding dangers posed by overconsumption and implications for health and longevity (*1*). As a psychotropic drug, ethanol severely affects cognition and behaviour, leading to intentional and accidental injuries, blunt trauma, and motor vehicle accidents requiring emergency hospital care (*2*, *3*).

Rapid and reliable laboratory methods for determination of EtOH in body fluids are important tasks for hospital and forensic laboratories worldwide. The results of toxicology testing in a clinical setting are likely to be used for medicolegal purposes. This happens when alcohol-related crimes are prosecuted or when people are injured in road traffic accidents and taken to a hospital emergency department for treatment, the measured EtOH concentration might later be used and interpreted in a legal context (*4*-*7*).

In medical diagnostics, enzymatic oxidation with alcohol dehydrogenase (ADH) is the method of choice for determination of EtOH in serum (Serum Alcohol Concentration, SAC) or plasma (Plasma Alcohol Concentration, PAC). The full automation of this procedure is ideal for emergency settings, because it can be performed 24/7 along with other routine clinical chemistry tests, in a high throughput workflow by laboratory personnel without specialized training or expertise (*8*).

Very often, SAC or PAC determinations are included as preliminary screening results on admission and later complemented and confirmed by a separate analysis of whole blood (Blood Alcohol Concentration, BAC). This latter analysis is usually performed by specialized personnel at forensic laboratories using gas-chromatography (GC), that is preferred in forensic science and legal medicine for it can separate EtOH from other volatile substances (*9*).

The enzymatic oxidation method currently used in hospital laboratories represents the culmination of over a century of research and development work touching on all the key stages of the transition from biochemistry to clinical and laboratory medicine. Hence, this review is aimed to highlight the intra-analytical and post-analytical aspects of EtOH determinations in serum and plasma and to provide an understanding of the *pros* and *cons* of the various methodologies available in a clinical environment. Hopefully, this will help to establish safe and effective operational procedures and boost confidence in the analytical results when used and interpreted for clinical and forensic purposes.

## Literature search

The scientific literature used to prepare this review was derived from various databases including Pubmed, Google Scholar and SCOPUS. We searched these databases up to December 2024 and no restriction was placed on language of the published articles.

**Figure 1:**
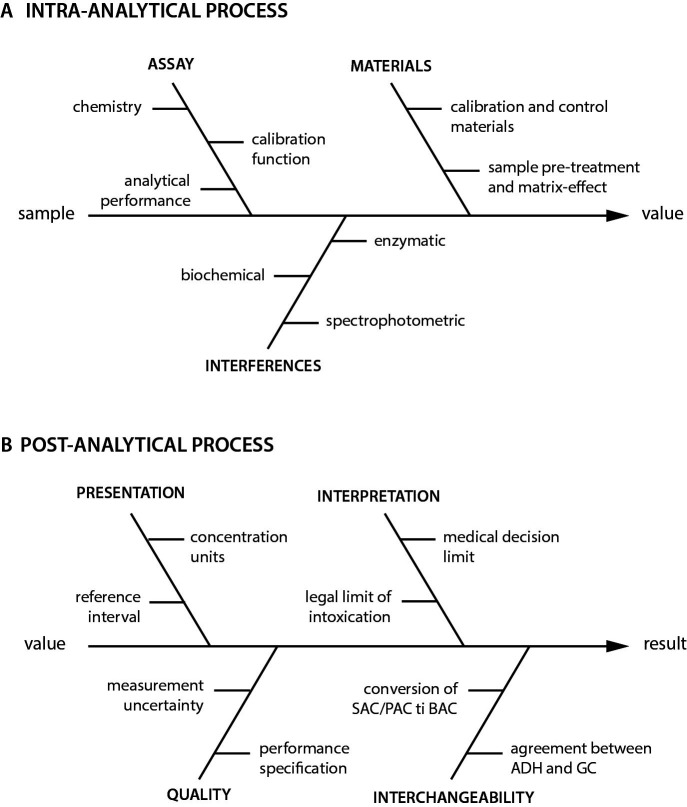
Factors contributing to the a) intra- and b) post-analytical phases in determination of ethanol in plasma (PAC) and serum (SAC) by the alcohol dehydrogenase (ADH) enzymatic method. The fish-bone diagram conceptualizes the inter-relationships between these factors within the analytical processes that formed the basis of our literature review. In particular, this illustrates how we refined a preconceived framework based on relevant intra- and post-analytical information gathered progressively through the literature review. GC - gas chromatography.

To develop a literature search strategy, we categorized the subject area based on post-analytical and intra-analytical aspects that influence the reliability of results, as illustrated in Figure 1. We then used various relevant keywords and phrases related to the determination of EtOH concentration in body fluids for clinical and forensic toxicology purposes. Notably, since no MeSH terms exist to indicate either the intra-analytical or post-analytical phases, we were unable to sufficiently narrow the searches. As a result, differently formulated queries often yielded redundant results or retrieved off-topic papers. Consequently, the articles retrieved from the initial database search were carefully reviewed, and their reference lists were examined in hopes of identifying relevant articles that had been missed, including those published before the 1990s, which may not be indexed in certain online databases.

Based on the title and abstract, 107 documents were initially deemed eligible for review, including journal articles, book chapters, symposium abstracts, conference proceedings, educational newsletters, white papers, and press releases. Of these, 62 were included in the present work due to their relevant content, while 5 could not be retrieved.

## Historical background

Chemical oxidation methods (see Appendix 1) were used successfully to measure EtOH in body fluids by the end of the 19^th^ century. However, the analysis was not specific for identification of EtOH, because other endogenous volatiles in blood (*e.g*., acetone), or ingested (*e.g.*, methanol and isopropanol), or inhaled (*e.g.*, diethyl ether) gave false high results. The isolation of pure crystalline alcohol dehydrogenase (ADH) in the 1950s provided a partial solution to this problem because of the milder oxidation conditions and development of EtOH-specific enzymatic methods (*10*-*12*).

In 1951, the first ADH-based methods were independently developed by two research groups that were interested in the enzymes involved with eukaryotic cell metabolism (*13*). The Swedish group was led by Roger Bonnichsen (1913-1989) and the later Nobel Prize in Medicine Hugo Theorell (1903-1982), while in Germany the researchers were T. Bücher and H. Redetzki (*14*, *15*). Quantitative analysis was done using the optical test developed earlier by the Nobel Prize in Physiology Otto H. Warburg (1883-1970) with his assistant W. Christian, who exploited the UV absorption peak of the reduced cofactor nicotinamide adenine-dinucleotide (NADH) to indirectly measure the amount of oxidized ethanol (EtOH) (*16*, *17*).

In 1957, the American company Technicon introduced the first fully-automated system for spectrophotometric enzymatic analysis: the continuous flow AutoAnalyzer (*18*). This paved the way for further development, in 1961, of the first reaction rate analyzer utilizing enzymatic oxidation with ADH (*19*). Just a year later, in 1962, H.V. Malmstadt published the first completely automated BAC assay using ADH as the reagent (*20*). This method, based on a non-equilibrium optical test developed earlier by Professor Keith Dalziel (1921-1994), managed to lower the assay time to just a few minutes (*21*). Research in this area continued to thrive throughout the decade (*22*-*25*).

However, by the 1970s, manual methods still dominated the commercial assays available to clinical and forensic laboratories (*26*, *27*). The “ALC” reagent pack, released by DuPont Instruments for its Automated Chemistry Analyzer (ACA), was a notable exception (*28*). The ACA, being more versatile, cost-effective, and simpler to use than the AutoAnalyzer, became a valuable tool in clinical toxicology and emergency medicine, providing rapid results with high sample throughput (*29*, *30*).

In the 1980s, the increasing use of gas chromatography (GC) in forensic applications helped solidify the role of the ACA and ALC in diagnostics (*31*). This period also marked the widespread adoption of SAC/PAC as the preferred matrix for clinical chemistry testing, thus avoiding interferences from hemoglobin when using spectrophotometric methods of analysis (see Appendix 2). By the 1990s and into the 2000s, further advancements focused on improving the specificity of the enzyme and refining procedures, which extended the use of commercial kits to analyze other biological matrices (*32*, *33*).

## Intra-analytics

### Chemistry of the assay

The current test methods utilize yeast ADH (alcohol:NAD oxidoreductase, EC 1.1.1.1) in a variable sensor-signal zero-order kinetic assay to measure EtOH concentrations typically ranging from 0.1 to 5.0 g/L (with variation depending on the manufacturer and instrument used) (*34*). Alcohol dehydrogenase, an oxidoreductase enzyme, catalyzes the oxidation of primary and secondary alcohol groups (R-OH and R_2_-CH-OH) in donor compounds, using NAD^+^ as the electron acceptor and zinc to coordinate substrate binding (*10*, *35*). The reaction is the following:

R.1: CH_3_CH_2_OH + NAD^+^ → CH_3_CHO + NADH + H^+^ (pH 9.2).

The enzyme’s substrate selectivity is influenced by its strain, and for yeast, it predominantly acts on primary saturated straight-chain alcohols (such as EtOH, n-propanol, and n-butanol) as well as saturated aldehydes like acetaldehyde (*35*, *36*). Table 1 shows the selectivity of enzymes used in some commercially available automated methods for routine testing of EtOH in serum and plasma.

**Table 1:** Percentage of substrate specificity of automated alcohol dehydrogenase (ADH) assays (tested at 1.0 or 2.0 g/L depending on the manufacturer) with ethanol = 100%

Substrate	ACA* (Du Pont)	Emit II Plus (Syva/Siemens)	REA Ethanol^†^ (Abbott)	Dimension Flex ETOH^§^ (Siemens)	ETOH2 (Roche)	DRI (Thermo Fisher)
	(%)					
Methanol	+ 0.4	< 1	< 1	< 1	< 1	< 1
n-Propanol	+ 67.3	+ 14.2	+ 63.6^ǂ^	+ 26.9	+ 8.0	+ 10.7
n-Butanol	+ 44.3	+ 3.7	+ 27.4	+ 4.2	+ 2.8	+ 1.7
Isopropanol	+ 12	< 1	< 5	< 1	< 1	< 1
Isobutanol	- 1.2					
Ethylene glycol	< 1	< 1	< 1	< 1		< 1
Propylene glycol		< 1	< 1	< 1		
Acetaldehyde		< 1		- 1	- 1.1	
Acetone	- 1.2	< 1	< 1	< 1	< 1	< 1
Acetic acid	- 1.5					
Lactic acid	- 1.2					
*First generation assay. ^†^With diaphorase. ^ǂ^Tested at 0.1 g/L. ^§^Tested concentration varies with the substance.						

At near-neutral pH, the reaction equilibrium (R.1) is shifted toward the left, particularly for yeast ADH, as its physiological role involves regenerating NAD^+^ equivalents for glycolysis (*36*). By adjusting the pH to more alkaline conditions (pH > 8.5, with an optimum of 9.0-9.2), the reaction equilibrium is shifted to the right (*14*, *37*). However, because the redox potential of the NAD^+^/NADH couple (E’0 = - 0.320 V) is relatively high, this makes it difficult to achieve complete oxidation of the EtOH. To address this problem, the acetaldehyde formed during the reaction must be removed by means of a “trapping” agent, such as semicarbazide or tris-(hydroxymethyl)-aminomethane, which drives the oxidation reaction to completion (*30*, *36*-*38*).

### Calibration function

In this kinetic method, a 1-point calibration (blank + concentrated calibrator) is employed, as described by the equation (Eq):

S = C × [A_s_(t_2_) – A_s_(t_1_)] / [A_c_(t_2_) – A_c_(t_1_)] (Eq. 1)

where: S is the unknown sample concentration, C is the nominal calibrator concentration, A_s_ and A_c_ represent the absorbance of the sample and calibrator, respectively, t_1_ and t_2_ are the times of absorbance measurement before the reaction reaches equilibrium (*34*).

This approach magnifies imprecision in quantification, compounded by the spectrophotometer’s inherent response characteristics (particularly background noise) and calibrator’s traceability (assigned *versus* actual concentration). As a result, the direction of the bias may vary depending on the positioning of the unknown sample relative to the calibration point - overestimating if below the calibration point and underestimating if above (*39*). This bias typically ranges within 5-10% and is completely eliminated by multi-point calibration (*32*, *33*).

### Analytical performance

The analytical performance is influenced by the relationship between the sample dilution in the reaction mix and the dynamic range of the spectrophotometer at 340 nm (*26*). In modern automated assays, these factors typically result in a linear range between 0.02-4.0 g/L (or alternatively 0.1-6.0 g/L, depending on the specific assay conditions) (*32*, *33*, *40*-*42*). Studies have shown that regardless of the estimation approach, the limit of detection (LOD) and limit of quantitation (LOQ) for SAC/PAC are approximately 5 mg/dL (0.05 g/L) and 10-20 mg/dL (0.1-0.2 g/L), respectively (*40*, *41*).

In single-center studies, the enzymatic assay demonstrates satisfactory repeatability and intermediate precision, with a coefficient of variation (CV) ≤ 5%, and good accuracy within 2.5% (*32*, *40*-*42*). This has been validated by the complete transferability of assay reagents across different instruments (*42*, *43*).

Proficiency testing/external quality assurance (PT/EQA) studies have shown a steady improvement in precision across generations of ADH assays, with the latest versions achieving performance levels comparable to GC (*44*-*46*). Despite this general trend, significant discrepancies remain both between and within manufacturers (*47*). These discrepancies are likely due to differences in automation technology/device configuration, as well as specific assay parameter settings, which result in distinct precision profiles across the measurement range (*46*, *47*).

### Spectrophotometric interferences

Spectrophotometric measurements are taken at a wavelength of 340 nm, where NADH exhibits its maximum absorbance peak, for highest sensitivity (*37*). Measurements can also be performed at wavelengths up to 366 nm or 383 nm to enhance selectivity and reduce background interference. This, however, results in a near 50% reduction in assay sensitivity (*36*, *48*). To correct for any inhomogeneity or turbidity in the reaction mixture, dual-wavelength (bichromatic) readings at 340 nm (reaction) and 505 nm (background) can be used (*49*).

Free oxygenated hemoglobin (Hb) exhibits a secondary UV absorption peak near 340 nm, making it the primary potential interfering substance in the assay. In contrast, bilirubin shows no significant absorbance in this region of the spectrum (*15*). In bichromatic assays with secondary wavelength for background correction set within the UV range (*e.g.*, 383-405 nm), there is a risk of “over-blanking” that causes false low responses (*30*). As a consequence, SAC/PAC is negatively biased by - 6% to - 16%, with the greatest effect at higher free Hb concentration (10 *vs.* 1 g/L) and lower SAC/PAC value (0.5 *vs.* 2 g/L) (*30*, *50*). Remarkably, over-blanking is observed across different analytical platforms and different generations of ADH assays (*30*, *50*-*52*).

Lipids, particularly triglycerides, exhibit strong absorption around 340 nm, which can lead to a positive bias and, in some cases, false positives (*i.e.*, SAC > 1.0 g/L) (*44*). However, no systematic study has been conducted to determine the threshold at which triglyceride concentration induces a positive bias or false positive, without any visible indication of turbidity (*53*). No significant UV interference from drugs has been reported to date (*26*).

In general, since this kinetic assay requires diluting the sample ~100 fold into the reaction mix, and current methods do not correct for background interference at UV wavelengths, the following interference thresholds are typically reported in datasheets: up to 800 mg/dL (8.0 g/L) of free hemoglobin, 30 mg/dL (513 μmol/L) of conjugated bilirubin, 60 mg/dL (1026 μmol/L) of unconjugated bilirubin, and 1000 mg/dL (11.4 mmol/L) of triglycerides (from synthetic lipid emulsion). However, actual performance may vary depending on the specific assay conditions (*52*, *54*).

### Biochemical interferences

Interference in the assay can arise from endogenous factors such as intoxication (*e.g.*, with alcohols like isopropanol) or metabolic conditions that increase the levels of potentially interfering substrates (*e.g.*, ketoacidosis) or alter NADH levels (*e.g.*, lactic acidosis).

Isopropanol intoxication has been reported to cause a biased SAC/PAC reading if either EtOH is present or if isopropanol alone is at concentrations greater than 1.5 g/L (indicative of severe intoxication) (*29*, *55*). However, these observations were based on DuPont ACA instrumentation, where the strain of ADH enzyme exhibited significantly higher cross-reactivity to isopropanol than current methods (see Table 1). Therefore, endogenous contamination from isopropanol is less of an issue with newer enzymatic assays, as is the case with other toxic alcohols (methanol and ethylene glycol) (*32*, *33*).

Acetone and other ketone bodies (acetoacetate and β-hydroxybutyrate), on the other hand, do not cause false positive results in the enzymatic method, regardless of their endogenous concentrations (*32*, *56*). This represents a major advantage of ADH methods compared with the earlier chemical oxidation, because in poorly treated diabetics blood-acetone concentrations are elevated. This lack of interference from acetone is consistent with the enzyme’s selectivity and the test conditions, which encourage ADH to act as an oxidizer rather than a reducer, preventing reactions with acetone and other ketones that might be present in the biological specimen.

### Enzymatic interference

**Figure 2:**
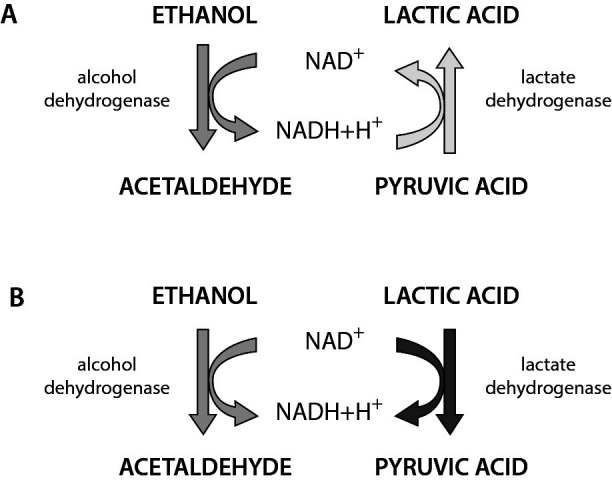
Balance of oxidized and reduced co-enzyme (NAD^+^ + and NADH + H^+^) in the oxidation of ethanol by alcohol dehydrogenase (ADH). In the cytoplasm of hepatocytes (a), lactate dehydrogenase (LD) consumes pyruvic acid (PA) to restore the NAD^+^ equivalents used by ADH to oxidize ethanol to acetaldehyde. In samples with elevated concentrations of LD and lactic acid (LA) (b), LD reduces LA to PA, producing NADH + H+ adding to that (if any) produced by ADH used in the assay, leading to a corresponding increase in absorbance at 340 nm.

Lactate dehydrogenase (LD) and lactic acid (LA) can potentially interfere with the assay of ethanol by enzymatic oxidation with ADH as illustrated in Figure 2.

In this case, excess NADH is produced non-specifically alongside EtOH oxidation, which can lead to a positive bias or even false positive results for EtOH (*57*, *58*). Depending on the assay, the spurious SAC/BAC can range from 0.2 g/L to 2.0 g/L (see Table 2). This biochemical interference is avoided by protein removal (acid precipitation or ultrafiltration) or LD inhibition (by oxamate) (*57*, *59*).

**Table 2:** Clinical cases of spurious *antemortem/perimortem* SAC/PAC with undetectable ethanol at GC analysis

**Ref.**	**Case**	**Age (yr)**	**Gd**	**Manufacturer**	**EtOH (g/L)**	**AST (U/L)**	**ALT (U/L)**	**LD (U/L)**	**LA (mmol/L)**	**Diagnosis**
(*67*)	1	6	M	Synchron Systems ETOH Alcohol (Beckman Coulter)	0.52	18,798	9170		> 20	Hypoxic-ischemic encephalopathy due to resuscitative drowing
	2	2	F		0.53	1404	2322			Natural death due to septicemia
(*66*)	1	67	M		0.545			17,802	14.6	Liver infiltrated (intravascular large B-cells lymphoma)
					0.493			16,534	20.3	
(*68*)	1	85	F	DRI Ethyl Alcohol Assay Microgenics (Thermo Fisher)	2.0			294		Medical history of aortic stenosis, ischemic heart disease, heart failure
					1.0			188		
(*61*)	1	33	M	Roche Ethanol Gen.2	2.0*	229	144			Car crash with minimal radiographic signs of organs damage
(*62*)	1			Bayer Advia 1650 Ethanol Assay (Siemens)	1.52^†^	8330	3540	8075	22.5	Hepatocellular necrosis due to acetaminophen ingestion
	2				1.52^†^	7080	12,286	4871	1.2	Hepatocellular necrosis due to acetaminophen ingestion
	3				1.56^†^	18,012	4119	10,147	5.1	Hepatocellular necrosis due to acetaminophen ingestion
(*60*)	1	75		Syva EMIT Alcohol Assay	0.17^ǂ^			2379	15.4	Chronic liver failure, severe metabolic acidosis, renal failure
	2	55			0.51^ǂ^			8015	12.3	End-stage liver disease
(*59*)	1		F	Syva EMIT Alcohol Assay	0.69			27,000	15	Cadaveric kidney transplant after diabetic end-stage renal disease
	2				0.44			24,623	5.6	Inferior wall myocardial infarction one week after carotid endarterectomy
*no GC confirmation but with normal anion gap. ^†^< 0.09 g/L with Dade-Behring RXL MAX. ^ǂ^< 0.010 g/L with Roche Ethanol Reagent. SAC - serum alcohol concentration. PAC - plasma alcohol concentration. GC - gas chromatography. Gd - gender. EtOH - ethanol. AST - aspartate aminotransferase. ALT - alanine aminotransferase. LD - lactic dehydrogenase. LA - lactic acid.										

Given that the zero-order kinetic assay typically involves ≈ 20-fold sample dilution and ≈ 200-fold ADH concentration, the original LD/LA concentrations in the sample must match the ADH/EtOH ratio in the reaction mix to produce notable interference (*60*, *61*). This makes the interference characteristically manufacturer-specific, because each assay kit has its own ADH and NAD^+^ concentration, as well as specific instrumental parameters (*60*, *62*). Thereby, some assays show a clear cutoff value of LD for spurious SAC/PAC interference, whereas others are completely insensitive to both LD and/or LA elevation (*63*, *64*).

Using the most recent automated assays, the interference from LD and LA typically requires that LD > 25,000 IU/L if LA > 14 mM or LD > 40,000 IU/L if LA > 4 mM (*59*, *60*). Therefore, it is not associated with either a specific pathology or a traumatic injury, but with the particular clinical conditions of the patient that cause the LD/LA to reach the required thresholds (*61*, *63*-*65*). Indeed, a number of reports show the interference to be associated with metabolic imbalance and organ failure (see Table 2) (*62*, *66*, *67*). Remarkably is some of them, spurious SAC/BAC results are observed even with slight LD/LA elevation, suggesting the involvement of other unknown factors in its genesis (*61*, *68*). A PT/EQA from the College of American Pathologists (CAP) conducted in 2011 showed that the enzymatic tests marketed in the United States exhibit large differences with respect to this interference (*69*). Unfortunately, no such information is still available for the European Union.

The release of catalase (CAT, EC 1.11.1.16) during hemolysis has been postulated as source of negative bias alongside over-blanking, as this enzyme is abundant in red blood cells (*51*). Catalase can utilize EtOH as a hydrogen donor *via* the hydroxyl group to decompose hydrogen peroxide (H_2_O_2_) in a reaction known as the “peroxidatic” reaction, even when H_2_O_2_ is not actively produced (*70*). This oxidation mechanism does not require nucleotide cofactors (or at most, consumes NADPH to protect itself from peroxidation), yet it efficiently metabolizes EtOH. Although it is a plausible cause for the negative bias observed in hemolyzed specimens, there is no experimental proof of its involvement. The only indirect evidence seems the mitigation of LD/LA positive bias as free Hb increases in the sample (compare values in Table II and III in reference (*60*) produced by the Syva assay method).

### Calibration and control materials

Enzymatic kits are typically provided with calibration and control materials, which are often based on water or a surrogate plasma/serum matrix. Usually, there is no bias correction used to determine the assigned value to calibrators (*33*, *71*).

While the nominal stability of these materials depends on the manufacturer, on-board stability plays a significant role in assay performance, as EtOH is a volatile compound that can evaporate from the specimen (*72*). When using sample cups for loading, a loss of less than 10% of the nominal EtOH concentration has been observed after 1 hour of resting uncovered and 2 hours when covered (*33*). Loss of EtOH is influenced by the ambient temperature, and this effect is more pronounced with small sample cups (0.2 mL) compared to regular ones (2 mL), and applies to both serum and plasma samples (*33*, *40*).

### Sample pre-treatment and matrix effect

Owing to the high dilution factor used in the reaction mix, pre-treatment of the sample is generally not necessary to remove interferences. However, acid precipitation (*e.g*., 1:10 dilution with 0.38 M perchloric acid or 1:2 dilution with freshly prepared 6% trichloroacetic acid) may be required to eliminate interference from hemoglobin and some enzymes, such as CAT and LD (*30*, *49*, *73*). This procedure demonstrates a recovery rate of 92-102% across a concentration range of 0.3-2.2 g/L, but it introduces a significantly negative bias when paired naïve samples are analyzed by either a reference GC method (≈ - 4%) or the same enzymatic method (≈ - 6%) (*26*, *49*, *73*, *74*). In this case, SAC/PAC measured in acid-treated matrix (supernatant) is about 5% higher than BAC (*73*).

Anticoagulants have the potential to interfere with enzymatic activity, as some can bind zinc ions essential for the reaction. However, in the case of ADH, the dilution factor likely explains why changing the sample matrix does not produce any observable effect (*26*). Specifically, heparin, sodium fluoride (with or without potassium oxalate), ethylenediaminetetraacetic acid (EDTA), and citrate do not cause any statistically significant bias (*42*, *75*).

## Post-analytics

### Concentration units

The analytical results of ethanol determinations in biological specimens can be expressed in various concentration units, depending on the context and international conventions. The most commonly used units are mass/volume (m/v), such as g/L (mg/mL) and mg/dL (mg/100 mL). Many clinical laboratories in EU countries prefer to use SI units of concentration (mmol/L), so it is necessary to account for EtOH’s molecular weight (MW = 46.05 g/mol) when converting weight of solute in grams or mg into moles or mmol. In some cases, particularly for forensic purposes, ethanol concentrations in blood are expressed in mass/mass (m/m) units. In this case, the solute concentration is slightly lower compared to m/v units, because the specific gravity (density) of blood is 1.055 g/mL and that of plasma/serum is 1.030 g/mL, respectively (*75*). Table 3 provides the calculations for unit conversion.

**Table 3:** Conversion formulas for ethanol concentration expressed in mass/volume units (*e.g.* g/L) to other concentration units (*e.g.* g/kg) including SI units

**mass/volume units**			**mass/mass units**				**SI units**
g/L	mg/dL	g/100 mL (% m/v)	specimen	g/kg (mg/g)	mg/100 g	g/100 g (% m/m)	mmol/L*
0.20	20	0.02	*wb^†^* *p/s^ǂ^*	0.095 0.097	9.5 9.7	0.0095 0.0097	2.17
0.3	30	0.03	*wb* *p/s*	0.284 0.291	28.4 29.1	0.0284 0.0291	6.51
0.5	50	0.05	*wb* *p/s*	0.474 0.485	47.4 48.5	0.0474 0.0485	10.85
0.8	80	0.10	*wb* *p/s*	0.758 0.777	75.8 77.7	0.0758 0.0777	17.36
1.5	150	0.15	wb *p/s*	1.422 1.456	142 146	0.142 0.146	32.56
*Molecular weight of ethanol = 46.07 g/mol. ^†^Density of whole blood (wb) = 1.055 g/mL. ^ǂ^Density plasma/serum (p/s) = 1.030 g/mL.							

### Reference interval

Ethanol can be produced endogenously during the detoxification of acetaldehyde generated by cellular metabolism, or intestinal dysbiosis (a phenomenon often referred to as “auto-brewery syndrome” (*76*, *77*). However, in healthy individuals, the corresponding endogenous ethanol concentration in peripheral blood is typically in the range of 10^-3^ g/L (0.001 g/L), a trivial amount. In conditions such as diabetes or liver cirrhosis, the endogenous ethanol concentration is usually only one or two orders of magnitude higher and lacks medico-legal significance (*78*-*80*). As a result, no specific reference range for endogenous EtOH exists, since such a range would likely be indistinguishable from the lower limit of quantification for both enzymatic and gas chromatographic methods (*80*).

Most laboratories prefer to use a practical analytical cut-off concentration of 0.1 g/L (2.17 mmol/L) before they report a patient’s BAC as being positive or not. A result below this cut-off would therefore be report as “ethanol not detected” and not as < 0.1g/L, which implies that there might have been a low concentration of ethanol in the specimen analyzed, which is not necessarily the case.

### Performance specifications

Performance specifications are essential for defining the acceptability of laboratory test results for diagnostic purposes. In the United States, acceptance limits (AL) for SAC/BAC are mandated by the Clinical Laboratory Improvement Amendments (CLIA). The initial CLIA issue in 1988 set the AL at ≤ ± 25% for SAC/PAC, while the most recent update in 2022 revised it to ≤ ± 20% (*81*). It is important to note that the AL represents the maximum allowable deviation from the target value, considering both bias and imprecision, and thus reflects the allowable total error (aTE).

In Europe, there is no official regulation issued by the European Union. However, at the national level, the only regulation is the “Richtlinien der Bundesärztekammer” (“Rili-BAEK”), which was issued in Germany by the German Federal Medical Council. In the Rili-BAEK, the AL is presented as the relative root mean square deviation (Δ). For SAC/PAC, the 2024 update provides separate values based on the assay result: Δ ≤ ± 9% for SAC/PAC ≤ 0.6 g/L and Δ ≤ ± 15% for SAC/PAC up to 5 g/L. For method comparisons, these values are adjusted to Δ ≤ ± 12% for SAC/PAC ≤ 0.6 g/L and Δ ≤ ± 21% for SAC/PAC up to 5 g/L (*82*).

To date, the European Federation of Laboratory Medicine (EFLM) has not yet provided analytical specifications regarding the enzymatic method of analysis in the Biological Variation Database.

### Measurement uncertainty

Measurement uncertainty (MU) is required to comply with ISO/IEC 15189 and ISO/IEC 17025, making it relevant for both clinical and forensic analysis. The top-down model is preferred for chemical analysis and, as such, is commonly used in laboratory medicine to calculate the MU of clinical tests. However, since different approaches can be employed to account for sources of uncertainty, the MU can vary significantly depending on the method used.

When MU is calculated according to the Eurachem/CITAC Guide CG4 (“Quantifying Uncertainty in Analytical Measurement”), the following sources of uncertainty are considered: a) repeatability of analysis (u_rep_), b) assay calibration (u_cal_), and c) sample stability (u_stab_). At the lower limit of intoxication (LLI) of 0.5 g/L, the expanded uncertainty for PAC/SAC is 8.46% (95% confidence level, coverage factor k = 2) (*83*).

Alternatively, when the Nordic countries’ technical report guide (“Nordtest”) is followed, sources of uncertainty are derived from long-term data on intermediate precision (u_Rw_) and bias (u_bias_), estimated from both internal and external quality control data. Under this approach, at the LLI of 0.5 g/L, the expanded uncertainty for PAC/SAC ranges between 13.12% and 19.74% (95% confidence level, coverage factor k = 2) (*84*, *85*).

Notably, PT/EQA studies show that nearly all commercially available automated assays exhibit acceptable MU at 0.5 g/L, which is a critical forensic decision level as it represents the LLI for driving in several countries (*47*).

### Medical decision level and legal limit of intoxication

Medical decision levels (MDLs) can be established based on toxicological threshold values related to the effects of acute EtOH intoxication on performance and behavior. A widely used relationship between BAC and signs and symptoms derives from “Dubowski’s seven stages of alcohol influence” (Table 4), and are based on dose-dependent neuro-physical responses to EtOH observed at corresponding BAC (*86*). However, due to significant inter-individual variability (*e.g.*, gender, genetics, drinking habits), MDLs should be considered as guidelines for the general population (*87*, *88*). Notably, individuals who develop central nervous tolerance to the effects of EtOH might not show the characteristic signs and symptoms despite them having a high BAC or PAC when examined (*89*, *90*).

**Table 4:** Medical decision levels (MDL) according to Dubowski’s stages of acute alcoholic influence/intoxication

**Blood Alcohol Concentration (g/L)**	**Stage**	**Clinical signs and symptoms**
0.1 - 0.5	Subclinical	Behavior nearly normal by ordinary observation
0.3 - 1.2	Euphoria	Mild euphoria, diminution of attention, judgment and control, beginning of sensory-motor impairment, loss of efficiency in finer performance tests
0.9 - 2.5	Excitement	Emotional instability, loss of critical judgment, impairment of perception, increased reaction time, reduced visual acuity, sensory-motor incoordination, impaired balance, drowsiness
1.8 - 3.0	Confusion	Disorientation, disturbances of vision and of perception of color, form, motion and dimensions, increased muscular incoordination, staggering gait, lethargy
2.5 - 4.0	Stupor	General inertia, markedly decreased response to stimuli, marked muscular incoordination, inability to stand or walk, vomiting, sleep or stupor
3.5 - 5.0	Coma	Complete unconsciousness, depressed or abolished reflexes, incontinence, Impairment of circulation and respiration, possible death
> 4.5	Death	Death from respiratory arrest
According to reference 31.		

Medical decision levels are also used to establish statutory legal limits of intoxication (LLI) in the workplace and when skilled tasks are performed, particularly concerning driving under the influence (DUI). In most countries, the statutory BAC limits for driving are set at 0.5 g/L (50 mg/dL), which is considered a threshold for impairment of sensory-motor functioning, leading to unsafe driving. However, the statutory BAC limits for driving range from 0.2 g/L to 0.8 g/L depending on the country (*91*). Special conditions might also apply, such as for novice and professional drivers, where a lower BAC is enforced and might be set as low as 0.1 g/L (so-called “zero-tolerance”) or at least undetectable with the usual analytical methods (*e.g.*, BAC < 0.01 g/L). Some countries enforce additional (higher) statutory BAC limits for driving representing a more serious traffic violation and higher penalties (BAC 1.0 g/L, 1.2 g/L or 1.5 g/L), thus reflecting the greater risk for a road-traffic crash and enhanced psycho-physical impairment associated with higher alcohol consumption (*92*).

### Agreement between ADH and GC

Based on regression analysis (either least-squares or Passing-Bablok, see Table 5) of SAC/PAC determinations by both automated and manual ADH assays compared with GC analysis, the results were highly correlated (r > 0.97) with only small systematic bias (< 6.0 mg/dL or < 0.06 g/L) (*26*, *32*, *33*, *40*, *41*). Remarkably, when multi-point instead of single-point calibration was adopted, perfect linear agreement was seen between ADH and GC methods without any statistically significant constant or proportional bias (*33*).

**Table 5:** Agreement between results of enzymatic alcohol dehidrogenase (ADH) method and gas chromatography applied to analysis of ethanol in serum/plasma

**Ref.**	**Method**	**GC**	**Matrix**	**N**	**r**	**Regression equation**	**Slope** **(95% CI)**	**Intercept** **(95% CI)**
(*26*)	Manual, Calbiochem “Alcohol Stat-Pack”	DI/FID	Serum	53	1.00*	PB*	1.00* (0.97 to 1.03)	0.06 g/L* (0.02 to 0.10)
(*33*)	Manual, Syva ETS-Plus Ethyl Alcohol Assay	HS/FID	Plasma Serum	95	0.99	OLS	0.92 (nr)	0.05 g/L^†^ (nr)
(*32*)	Manual, Syva ETS-Plus Ethyl Alcohol Assay	DI/FID	Serum	92	0.97	OLS	1.02 (0.97 to 1.07)	0.04 g/L (- 0.07 to 0.15)
(*40*)	Automated, Syva Emit Ethyl Alcohol Assay	DI/FID	Plasma	30	0.99	OLS	1.01 (nr)	0.06 g/L^ǂ^ (nr)
(*65*)	Automated, Ethyl Alcohol Flex Reagent Cartridge	DI/FID	Plasma Serum	24	0.98*	PB*	0.99* (0.89 to 1.06)	0.06 g/L* (- 0.06 to 0.23)
(*41*)	Manual (?), Thermo Fisher Scientific	HS/FID	Serum	30	1.00	PB	0.97 (nr)	0.02 g/L^ǂ^ (nr)
*calculated from published data. ^†^converted from the result published in g/dL. ^ǂ^converted from the result published in mg/dL. GC - gas chromatography. DI - direct injection. FID - flame ionization detector. PB - Passing-Bablok regression. OLS - ordinary least squares regression.								

In PT/EQA studies, which reflect the combined effect of pre- and intra-analytical factors, ADH and GC show good agreement in SAC/PAC determinations for both accuracy and precision, regardless of the generation of ADH assay and the GC method (headspace or direct injection, packed or capillary column) (*44*, *45*). When measuring the degree of deviation from PT’s target value, the z-score obtained with ADH and GC do not statistically differ proving that method is not a factor for SAC/PAC determination (*93*).

### Conversion of SAC/PAC to BAC

The accuracy of the factor used to convert SAC/PAC to BAC depends on differences in water content and cellular components between the biological matrices (*94*). As a result, a single SAC/PAC value may correspond to a range of BAC values (*95*). The conversion factor used is therefore typically an average or median value derived from population data (*94*). It is important to note that this prediction interval is based on empirical data, meaning its reliability is directly tied to the size of the sample used to generate the results (*96*).

Typically, BAC is measured chromatographically, while SAC/PAC is determined enzymatically. This difference introduces a small bias in the conversion factor, so that SAC/PAC to BAC ratio often varies depending on the prevailing BAC concentration (*97*, *98*). This is likely to be caused by differences in imprecision - primarily from handling distinct matrices - pipetting and calibration parameters in the methods of measurement.

**Figure 3:**
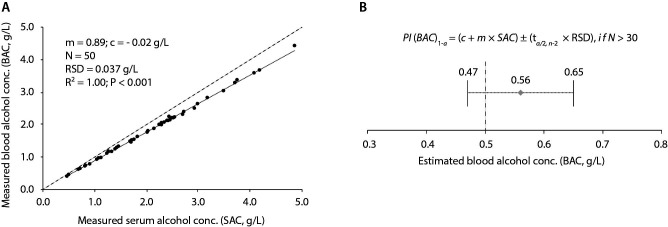
Example of the conversion of serum alcohol concentration (SAC) to blood alcohol concentration (BAC) using linear regression and estimation of the prediction interval (PI). The calculations refer to the data reported by Winek and Carfagna (*95*). Panel a: Ordinary least-squares (OLS) regression analysis was used to create a relationship between the measured SAC values (independent variable) and BAC (dependent variable), where m = slope, c = intercept and RSD is residual standard deviation. Panel b: shows the 95% PI for the estimated BAC corresponding to a measured SAC, assumed in the example to be 0.65 g/L. For a Type I error probability of 5% (α = 0.05) the PI has a coverage probability of 95% (1-α = 0.95). Substitution into the regression equation (panel a) the PI (for N > 30) yields: PI(BAC)0.95 = (- 0.0152 + 0.885 x 0.65) ± (2.314 x 0.037) = 0.56 ± 0.090 g/L corresponding to 0.47 to 0.65 g/L (panel b).

Another approach would be to use a regression function for converting plasma or serum concentrations of ethanol into BAC (whole blood), rather than relying on a single conversion factor (*98*). An example is provided in Figure 3.

## Discussion

Automated enzymatic methods for the determination of ethanol in plasma and serum are widely accepted in emergency medicine and toxicology, because they produce rapid and accurate results with acceptable diagnostic reliability. The ADH derived from yeast is highly specific for identification of ethanol and this avoids cross reactivity and interferences (see Table 5), which often occurs with immunochemical methods used in clinical toxicology. Therefore, the only significant source of bias stems from difference between SAC/PAC and BAC, which is however irrelevant for the MDLs. Besides, analytical automation, together with the conditions of non-equilibrium of the reaction, ensures low imprecision and a MU that meets legal requirements for diagnostic tests.

However, despite these important features, this literature review has identified some issues not mitigated by the analytical automation and the industrial production of reagents. In fact, when results of the published PT/EQA studies are evaluated (unfortunately few), it is evident that despite decades of use in the clinical laboratory, the ADH method is far from being standardized; there is no full interchangeability of reagents and methods, even within platforms of the same manufacturer (*46*, *47*).

Besides standardization, interferences, which are inherent to the chemistry of the assay, remain the greatest challenge to absolute clinical and forensic reliability. One example is interference from LD/LA and this is unfavorable both clinically and forensically: it cannot be macroscopically anticipated as with hemolysis, and is not related to the pathology itself but rather to how it manifests in the specific patient in terms of circulating LD and LA concentrations. This can lead to apparent measurement of EtOH in the sample when in fact not exists. Therefore, it can complicate the correct clinical assessment of the patient, but also potentially lead to the erroneous identification of cases of deliberate or negligent alcohol intoxication (*68*).

Interference with the ADH method occurs if there is free Hb in the sample (*e.g.* if hemolysis has occurred), because this causes a negative bias and can bring the SAC/BAC value below the LLI threshold. Although this is less relevant at the diagnostic level (see the TEa), it becomes significant when the ADH method is used as a screening tool in targeted forensic toxicology investigations (for example, driving under the influence of alcohol) to screen for positive samples that require confirmatory analysis by GC. In this case, a variation of 6% (the minimum observable) can bring the SAC/BAC value below the current LLI threshold (which triggers the subsequent GC analysis) if MU is not taken into account.

Since clinical analyses always take precedence over forensic ones, depending on the critical condition of the patient, the result of PAC or SAC determined by the ADH method might be the only available data for use as forensic evidence. Alternatively, medical treatment of the patient might delay the collection of forensic samples to the point of causing a significant change in the concentration of ethanol by oxidative metabolism in the liver at a rate of 0.15 g/L/h on average. Furthermore, infusion of 3 liters of fluids during hypovolemic shock was found to cause up to 7-10% negative bias of the determined SAC (*99*).

In forensic cases, the clinical data can be highly questionable if confirmation analysis by GC is not possible. This is especially true if, as may occur in the aforementioned critical conditions, the sample volume collected for diagnostics is small (because it was partially used along with clinical chemistry tests), or was not preserved by the laboratory due to lack of appropriate communication.

Efforts to strengthen the reliability of the ADH method, particularly in the forensic setting, such as when SAC and PAC are converted to BAC, requires a detailed examination of the laboratory procedures used to generate the results. In this regard, Table 6 illustrates some key-elements and future strategies that can take advantage of total laboratory automation (TLA). As it can be seen, they are not much more complicated than the proper use of evacuated blood sampling tubes, which is necessary to ensure forensic reliability in BAC pre-analytics (*72*).

**Table 6:** Strategies and operative procedures to improve the diagnostic validity and forensic defensibility of clinical results by the enzymatic alcohol-dehydrogenase (ADH) method

**Interference**	**Effect**	**Action**
Lactate Dehydrogenase/ Lactic Acid	Positive bias (clinical) and false positives (forensic)	‘Secure’ storage regime: with modern total laboratory automation (TLA) systems, this can be achieved by setting up an automatic aliquoting of the sample upon check-in; the special aliquot is sealed and stored, and can only be recalled for analysis with a password – remembering, of course, that in TLA all steps are tracked. “Add-on testing”/”reflex testing” routines: it can be implemented in the laboratory information system (LIS) or the middleware if TLA; in the presence of specific analytes in the emergency panel (*e.*g., AST and ALT as in Table 2), the LD/LA analysis is activated on the same aliquot to exclude enzymatic bias; if LD/LA values exceed the cut-off for the assay in the analytical platform, the SAC/PAC is not released with warning of mandatory GC confirmation
Free Oxygenated Hemoglobin	Negative bias (clinical) and false negatives (forensic)	“Add-on testing”/”reflex testing” routines: samples with an index of hemolysis above the acceptable is automatically placed on the GC confirmation list
SAC - serum alcohol concentration. PAC - plasma alcohol concentration. GC – gas chromatography. AST - aspartate aminotransferase. ALT - alanine aminotransferase. LD - lactic dehydrogenase. LA - lactic acid.		

In conclusion, the ADH method for analysis of EtOH in a laboratory setting does not meet forensic standards but can nevertheless be integrated into a robust procedure without compromising forensic usefulness and high-volume diagnostic testing. Of course, addressing the technical challenges necessary to improve the industrial standardization of commercial products is an achievable goal, but to be effective, it requires pressure from forces outside the laboratory. In our opinion, the enzymatic method, which has been a successful prototype in transforming numerous basic research ideas into a single diagnostic tool, is now ready to initiate a new phase: one where evidences derived from PT/EQA data can drive the improvement of the commercial assays.

## Data Availability

The data generated and analyzed in the presented study are available from the corresponding author on request.

## Appendix 1 – Determination of EtOH in whole blood by chemical oxidation

The chemical oxidation method uses potassium dichromate solubilized in sulfuric acid (*i.e*., Laconte’s solution) to detect EtOH, after it has been separated from biological matrices through aeration, distillation, or diffusion. The stoichiometry of the reaction is shown below:

R.2: 3CH_3_CH_2_OH + 2K_2_Cr_2_O_7_ + 8H_2_SO_4_ → 3CH_3_COOH + 2K_2_SO_4_ + 2Cr_2_(SO_4_)_3_ + 11H_2_O

R.2 is an “oxidimetric (redox) reaction” that measures EtOH indirectly through some by-product of its oxidation, in this case the reduction of Cr VI (K_2_Cr_2_O_7_) to Cr III (Cr_2_(SO_4_)_3_). Note that in the enzymatic method, which stems directly from the chemical method, the oxidimetric reaction uses an enzyme (ADH) as the catalyst and the by-product for quantitative analysis is represented by the NADH.

This oxidation of ethanol to acetaldehyde was discovered in the 1830s in Germany by the famous chemist Justus von Liebig (1803-1873) during his research on “animal fermentation” (*100*). Applications of the method to determination of ethanol in the human body began later, in the second half of the 19th century, particularly in industrialized nations like Germany, England, and France countries where social and toxicological issues caused by excessive drinking and drunkenness were causing problems for public health (*101*, *102*).

Maurice Nicloux (1873-1945) in France pioneered the quantitative analysis of BAC in alcoholics, using Liebig’s distillation apparatus (a cumbersome and bulky glassware device) (*103*-*105*). In the 1920s Erik M.P. Widmark (1889-1945) in Sweden introduced a micro-diffusion-based portable apparatus (a forerunner of point-of-care testing devices), making blood-alcohol testing practical and reliable enough to be used also in a legal context (*106*-*108*). Widmark’s outstanding work laid the foundation for modern forensic toxicology of EtOH (*109*).

## Appendix 2 – Determination of EtOH in whole blood by enzymatic oxidation

In order to determine BAC by enzymatic oxidation, it is necessary to remove plasma proteins and/or colored elements, from the biological matrix by treatment with acids (like Folin’s “unlaked blood”) (*36*, *110*). When this was done the results were comparable to those obtained by direct analysis of SAC/PAC (LOD = 0.007 g/L, LOQ = 0.024 g/L), although the sample pre-treatment causes small losses of EtOH in the absence of an internal standard (*49*). When indirect BAC methods were compared with GC methods the results showed a negative bias (systematic - 0.003 g/L and proportional - 5%), which makes this approach comparable in inaccuracy to the conversion of SAC/PAC to BAC (*49*, *74*).

In order to allow more direct measurement of BAC, the procedures have been modified by altering the composition of the analytical reagents used to determine SAC/PAC and/or the use of detection systems other than UV spectrophotometry.

Fluorimetry offers a way to directly measure BAC without any manipulation of the whole blood matrix (*25*). It uses the characteristic excitation and emission wavelength (340 nm and 460 nm, respectively) of NADH to monitor its rate of formation (*25*, *111*). Because of the presence of other fluorescent compounds, a significant background signal arises from whole blood, limiting BAC to LOQ ≥ 0.125 g/L and linearity up to 2 g/L (*111*).

The Radiative Energy Attenuation (REA) method developed by Abbott Laboratories represents the only fluorimetry application that was produced commercialy for diagnostics, first for stand-alone fluorimeters and then for discrete analyzers (*112*, *113*). This technique is a hybrid between colorimetry with reduction-oxidation dyes (*e.g.*, iodonitrotetrazolium, INT), used to determine the activity of NAD-dependent dehydrogenases, and fluorimetry based on fluorescein measurement.

With the REA method, dihydrolipoamide dehydrogenase or diaphorase (EC 1.6.4.3) oxidizes NADH to NAD^+^ and at the same time stoichiometrically reducing INT to formazan-INT. The formazan-INT has an absorption peak at 492-505 nm, which overlaps with the emission peak of fluorescein. Therefore, when fluorescein is excited, the fluorescence produced is attenuated in proportion to the concentration of formazan-INT, and in direct proportion to the EtOH concentration (*113*). Abbott Laboratories later improved their REA method using thiazoyl blue (MTT) to reduce the background signal (*71*).

The analytical performance of REA for determining SAC and BAC has compared well in several evaluation studies (*71*, *114*). The method requires only 0.05 mL of sample and the analysis time is about 5 minutes, with a linearity range from 0.01-3.0 g/L involving a six-point non-linear calibration (*115*). When direct comparisons were made between REA and GC analysis of the same specimens, neither a systematic nor a proportional bias was evident (*116*). Therefore, the REA method was evaluated and approved for the determination of EtOH in forensic laboratories, although it was not completely devoid of false positives (*71*, *117*).

The decline in the use of REA methods of analysis is linked to the longevity of proprietary clinical chemistry platforms (TDx, ADx, AxSYM), as well as the availability and spread of advanced GC technology from forensic laboratories to clinical laboratories.
